# Cell non-autonomous effect of hepatic growth differentiation factor 15 on the thyroid gland

**DOI:** 10.3389/fendo.2022.966644

**Published:** 2022-08-15

**Authors:** Seonhyang Jeong, Seul Gi Lee, Kook Hwan Kim, Xuguang Zhu, Woo Kyung Lee, Hwa Young Lee, Sunmi Park, Myung-Shik Lee, Sheue-Yann Cheng, Jandee Lee, Young Suk Jo

**Affiliations:** ^1^ Department of Internal Medicine, Yonsei University College of Medicine, Seoul, South Korea; ^2^ Department of Surgery, Daejeon Eulji Medical Center, Eulji University, Daejeon, South Korea; ^3^ Severance Biomedical Science Institute, Yonsei University College of Medicine, Seoul, South Korea; ^4^ Laboratory of Molecular Biology, Center for Cancer Research, National Cancer Institute, National Institutes of Health, Bethesda, MD, United States; ^5^ Department of Surgery, Open Nanotechnology Biotechnology Information technology Convergence Technology Research Laboratory, Severance Hospital, Yonsei Cancer Center, Yonsei University College of Medicine, Seoul, South Korea

**Keywords:** mitochondria, mitochondrial stress, mitokine, GDF15, thyroid cancer

## Abstract

The thyroid gland plays an essential role in the regulation of body energy expenditure to maintain metabolic homeostasis. However, to date, there are no studies investigating the morphological and functional changes of the thyroid gland due to mitochondrial stress in metabolic organs such as the liver. We used data from the Genotype-Tissue Expression portal to investigate RNA expression patterns of the thyroid gland according to the expression of growth differentiation factor 15 (GDF15) such as the muscles and liver. To verify the effect of hepatic GDF15 on the thyroid gland, we compared the morphological findings of the thyroid gland from liver-specific GDF15 transgenic mice to that of wild type mice. High GDF15 expression in the muscles and liver was associated with the upregulation of genes related to hypoxia, inflammation (TGF-α *via* NFκB), apoptosis, and p53 pathway in thyroid glands. In addition, high hepatic GDF15 was related to epithelial mesenchymal transition and mTORC1 signaling. Electron microscopy for liver-specific GDF15 transgenic mice revealed short mitochondrial cristae length and small mitochondrial area, indicating reduced mitochondrial function. However, serum thyroid stimulating hormone (TSH) level was not significantly different. In our human cohort, those with a high serum GDF15 level showed high fasting glucose, alanine transaminase, and alkaline phosphatase but no difference in TSH, similar to the data from our mice model. Additionally, high serum GDF15 increased the risk of lymph node metastasis to lateral neck. The hepatic GDF15 affected thyroid morphogenesis *via* a TSH-independent mechanism, affecting aggressive features of thyroid cancers.

## Introduction

Mitochondria, a cellular powerhouse, are involved in various cellular functions, such as signaling and differentiation. Mitochondrial dysfunction is often regarded as a tool for measuring senescence and is closely associated with metabolic disorders such as insulin resistance in skeletal muscles, liver, fat, heart, and pancreas (1–6). Impaired mitochondrial β-oxidation is observed in patients with nonalcoholic fatty liver disease (NAFLD), a cause of hepatic steatosis and liver fibrosis (7–10). In adipocytes, mitochondria perform lipogenesis by synthesizing triglycerides and are responsible for lipolysis *via* β-oxidation of fatty acids (6, 11). Further, mitochondrial dysfunction is tightly associated with decreased fatty acid oxidation, which results in insulin resistance, obesity, and diabetes (6, 12, 13).

Recent research has suggested that mitochondrial stress may be a bridge between mitochondrial dysfunction and metabolic diseases (14). In fact, novel mitochondrial stress-induced cytokines (mitokines) such as fibroblast growth factor 21 (FGF21) and growth differentiation factor 15 (GDF15) have been discovered while elucidating the role of mitochondrial stress (15, 16). Among those mitokines, GDF15, first identified as macrophage inhibiting cytokine-1, is a well-known biomarker of cellular stress (17, 18). GDF15 is also induced by mitochondrial stress, and is associated with various diseases such as inflammation, cancer, cardiovascular disease, mitochondrial disorders, chronic liver disease, and obesity (19–24). Mechanistically, GDF15 acts *via* a specific hindbrain (area postrema) glial-derived neurotrophic factor (GDNF) receptor alpha-like (GFRAL) to regulate energy homeostasis. Experimental GDF15 injection to animal models of obesity and diabetes have reported positive results (25–27). Likewise, liver specific GDF15 transgenic (GDF15 Tg) mice have a better metabolic phenotype. For example, GDF15 Tg mice showed lower body weight, lower fasting blood glucose and resistance to fatty liver disease compared to control mice (28).

The thyroid gland is an essential endocrine organ that maintains energy homeostasis in both mice and humans. Thyroid hormone synthesis is increased by thyrotropin-releasing hormone neurons that interact with leptin against excessive body energy accumulation, for instance, in case of obesity (29). Accordingly, metabolic diseases may be accompanied by the inactivation of these homeostatic mechanisms in the thyroid gland (30). Thus, in this study, we investigated the anatomical and functional effects of GDF15 on the thyroid gland. Consequently, we aimed to understand the effect of mitochondrial stress response in metabolic organs on the endocrine organs. To achieve our goal, we used data from Genotype-Tissue Expression (GTEx) portal and liver-specific GDF15 transgenic (GDF15 Tg) mice. Finally, for validation, we generated a human cohort and analyzed human phenotypes according to high serum GDF15 concentration.

## Materials and methods

### Transgenic mice

Liver-specific GDF15 Tg mice were generated as described in a previous study (28). Briefly, these mice were generated by microinjection of linearized pLiv7-GDF15 into C57BL/6 zygotes (Macrogen Inc., Seoul, South Korea). The mice used in the experiment were 28 week-old males. All animals used in the experiments were maintained in a specific pathogen-free facility accredited by the Association for the Assessment and Accreditation of Laboratory Animal Care International. The animal experiments were approved by the Institutional Animal Care and Use Committee of Yonsei University Health System.

### Patients

We generated a retrospective cohort of patients (N = 162, mean age = 42.8 ± 11.9) with conventional papillary thyroid carcinoma (PTC) underwent thyroidectomy at Yonsei Cancer Center from April to October 2010 (Seoul, South Korea). The clinical and biochemical parameters were retrospectively collected. All patients donated their blood for research purposes (GDF15 measurement), and the serum was stored at -80°C. This study has been approved by the Institutional Review Board of Severance Medical Center (Seoul, South Korea) on 30 September 2013 (No. 4-2013-0546); the requirement for informed consent was waived because of its retrospective nature of the study.

### Hematoxylin and eosin staining and Immunohistochemistry

Thyroid tissues were collected and fixed immediately in 10% formalin solution, and then made into paraffin-embedded blocks. Formalin-fixed paraffin-embedded tissue sections (4 μm thick) were stained with hematoxylin and eosin (H&E). Tissue sections were deparaffinized in Histo-Clear II (HS-202; National Diagnostics Inc., Charlotte, NC), rehydrated through a graded series of ethanol solutions, and then stained sequentially with hematoxylin (HHS16; Sigma-Aldrich, Munich, German) and eosin (HT110132; Sigma-Aldrich). To identify the thyroid isthmus, whole paraffin blocks of each specimen were cut into slices between 4~5 μm in thickness, and these were used for H&E. IHC staining was conducted using paraffin-embedded tissue sections (IHC-P). Briefly, sections were stained with antibody specific for GFRAL (AF5728, Agilent Technologies, Santa Clara, CA), mTOR (#2983, CST, Danvers, MA), p-mTOR S2448 (sc-293133), p-mTOR S2481 (sc-293132, Santa Cruz Biotechnology, CA, USA) were used according to the manufacturer’s protocols. Stained slides were scanned and analyzed by an Aperio Scanscope AT Turbo instrument and Imagescope software (Aperio Technologies, Vista, CA), respectively. The total size of the thyroid gland was quantified and analyzed using the area function of Image J, and protein expression was quantified using the Color Deconvolution2 plugin in Image J (31, 32). Staining intensities were averaged by randomly selecting 3 fields in each slide.

### Transmission electron microscopy

TEM was performed with the traditional methods. Briefly, tissue samples were fixed 12 hours in 0.1M phosphate buffer (pH 7.4) containing 2% glutaraldehyde, 2% paraform-aldehyde, and 0.5% CaCl2. After washing for 2 hours with 0.1 M phosphate buffer, the samples were treated with 1% osmium tetraoxide for 1 hour. Samples were dehydrated through a series of alcohol concentrations (50, 60, 70, 80, 90, 95 and 100%) and run for 10 minutes at each concentration. Tissue samples were embedded with a Poly/Bed 812 kit (Polysciences, Warrington, PA), polymerized in an electron microscope oven (TD-700, DOSAKA EM CO., LTD, Kyoto, Japan) at 65°C for 12hr. The block is equipped with a Diamond Knife in the Ultramicrotome (Leica EM UC-7, Leica Microsystem, Vienna, Austria), and is cut into 200nm semi-thin section and stained with toluidine blue for observation of optical microscope (Olympus Corp., Tokyo, Japan). The region of interest was then cut into 80nm thin sections using the ultramicrotome, placed on copper grids, double stained with 3% uranyl acetate for 30min and 3% Lead citrate for 7min staining, and imaged with a transmission electron microscopy (JEM-1011, JEOL, Tokyo, Japan) at the acceleration voltage of 80kV equipped with a Megaview III CCD camera (Olympus Soft Imaging Solutions, Lakewood CO). Measurement of mitochondrial major axis length was performed in at least five mitochondria per cell, in a minimum of 50 cells/experiment (250 mitochondria/experiment). Mitochondrial length was then quantified using the ROI Manager plug-in of Image J software (github.com/imagej/imagej).

### GDF15 and thyroid stimulating hormone measurement

Serum samples were aliquoted prior to storage at −80°C. Only one aliquot was retrieved for each assay to avoid multiple freeze/thaw cycles. Serum GDF15 level was determined using Human GDF-15 Enzyme-Linked Immunosorbent Assay (ELISA) Kit (ELH-GDF15, RayBiotech, Peachtree Corners, GA). For each assay, serum was diluted 1:5–1:500 into sample diluent, and duplicate assay was performed for each sample. Serum TSH was measured using a specific mouse TSH radioimmunoassay by previously described method using the RIA kit purchased from the National Hormone and Peptide Program (33, 34).

### Functional enrichment analysis using GTEx

GTEx project aims to provide resources to study the relationship of the regulation of human gene expression with genetic variation. All data related to gene expression levels was generated using disease-free ‘normal’ tissues, validated by histological examination. We used GTEx RNA-seq data from 46 kinds of human tissues (dbGaP Accession phs000424.v8.p2) to investigate GDF15 expression (35). Using these datasets, the expression of GDF15 was confirmed in eight human tissue types: kidney (n=85), adipose-subcutaneous (n=663), liver (n=226), thyroid (n=653), skeletal muscle (n=802), hypothalamus (n=97), whole blood (n=755), and heart-left ventricle (n=430). We also performed gene set enrichment analyses (GSEA) provided by the Broad Institute (36). The expression profile was compared and analyzed between the upper and lower quarters according to GDF15 expression. Enrichment Score (ES) values can be calculated using weighted Kolmogorov-Smirnov statistics. We selected significant gene sets based on the p-value ≤ 0.05 and the False Discovery Rate (FDR) q-value < 0.25.

### Statistical analysis

Statistical significance was determined using parametric Student’s t-test and non-parametric Mann-Whitney U-test. Group comparisons were performed using a 2-tailed chi-square test, Fisher’s exact test, or linear-by-linear associations, accordingly. Pearson’s correlation coefficient and Spearman’s rank correlation coefficient were used to investigate the association of clinical factors. Univariate analysis was performed using the log-rank test, and multivariate analysis was performed using the Cox proportional hazard model to identify independent predictors of high serum GDF15 level. All analyses were performed using IBM SPSS statistics 23.0 (SPSS Inc., Chicago, IL) and Prism (GraphPad Soft-ware, San Diego, CA). All results were considered statistically significant when P < 0.05.

## Results

### High GDF15 expression was related to upregulation of specific genes

**Figure 1:**
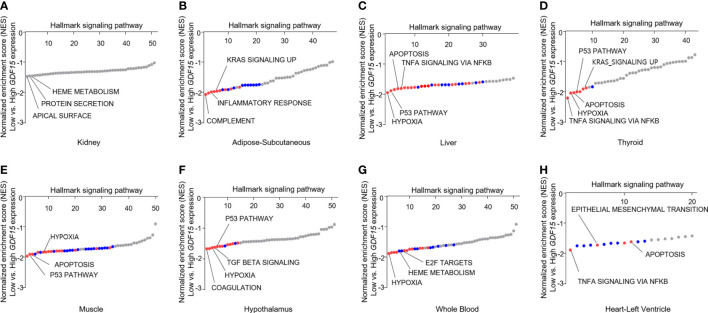
Functional analysis of gene expression data according to GDF15 expression. Representative results of gene set enrichment analysis (GSEA) using gene ontology “biological process” enrichment terms in tissues according to GDF15 expression (low vs. high GDF15 expression groups) for **(A)** kidney (n = 21 for each group), **(B)** adipose-subcutaneous (n = 165 for each group), **(C)** liver (n = 56 for each group), **(D)** thyroid (n = 163 for each group), **(E)** skeletal muscle (n = 200 for each group), **(F)** hypothalamus (n = 24 each group), **(G)** whole blood (n = 188 for each group), and **(H)** heart-left ventricle (n = 107 each group). Representative gene sets are indicated by gene set names. Red circles indicate significant gene sets (nominal P value ≤ 0.0001 and false discovery rate (FDR) q-value ≤ 0.25). Blue circles indicate gene sets with high FDR q-values although the P value is meaningful (nominal p-value ≤ 0.01 and FDR q-value > 0.25).

To understand the expression status of GDF15 in various organs, we analyzed the median expression values in 46 types of human tissues from GTEx. Kidney cortex showed highest expression of GDF15 mRNA (6.85 ± 3.35 log2(TPM+1)), whereas brain amygdala indicated lowest expression (-1.51 ± 2.36 log2(TPM+1)) (**Supplementary Figure 1A**). Metabolic organs such as subcutaneous of adipose (3.51 ± 1.89 log2(TPM+1)), liver (2.78 ± 2.34 log2(TPM+1)), and thyroid (2.66 ± 1.70 log2(TPM+1)) showed similar expression values. Overall, brain tissues showed relatively low GDF15 expression and like other brain tissues, hypothalamus (-0.70 ± 1.48 log2(TPM+1)) indicated low expression. In addition, whole blood (-0.91 ± 1.68 log2(TPM+1)), muscle (-0.07 ± 2.13 log2(TPM+1)) and heart left ventricle (-1.28 ± 1.79 log2(TPM+1)) also showed relatively low expression. Among 46 types of human tissues, we selected some types of tissues to perform GSEA based on functional importance in metabolism and GDF15 expression pattern (**Figures 1A–H**). GSEA using highest and lowest quartile according to GDF15 expression in kidney cortex provided significantly up-regulated gene sets (**Figure 1A**) such as apical surface (False Discovery Rate (FDR) q-value = 0.90, nominal (NOM) p-value = 0.008), Protein secretion (FDR q-value = 0.50, NOM p-value = 0.014) and Heme metabolism (FDR q-value = 0.34, NOM p-value =0.02). But, all FDR-q values were above 0.25, indicating not statistically meaningful enrichment. However, metabolic organ including subcutaneous adipose tissue, liver, muscle and hypothalamus showed coordinately enrichment of genes related to inflammatory response, TNFα signaling *via* NFκB, hypoxia, apoptosis, p53 pathway and TGFβ signaling. Interestingly, thyroid, whole blood and heart left ventricle also showed coordinately enrichment of genes related to TNFα signaling *via* NFκB, hypoxia, apoptosis, and p53 pathway.

Our GSEA consistently presented that high GDF15 expression is related to inflammation, hypoxia, apoptosis in these organs and to oncogenic signals in some tissues (KRAS signaling in subcutaneous adipose tissue and thyroid and epithelial mesenchymal transition in heart left ventricle, **Figures 1B–H**). Although genes related to mitochondrial dysfunction were not enriched directly in contrast to our postulation, heme metabolism, hypoxia and apoptosis might be related to mitochondrial function.

### High GDF15 expression in blood, muscle and liver was related to the expression of specific genes in thyroid glands *via* common and organ specific manner

**Figure 2:**
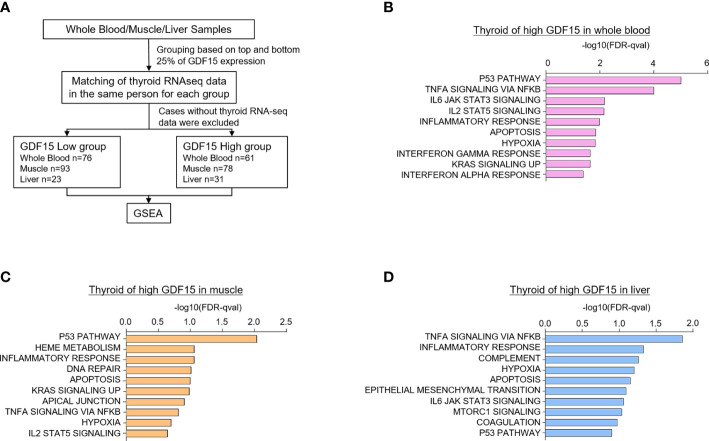
Upregulated gene sets of the thyroid gland according to high GDF15 expression of whole blood, muscles, and liver. **(A)** Schematic diagram of the analysis algorithm. **(B–D)** Representative upregulated gene sets according to high GDF15 expression of whole blood **(B)**, muscle **(C)**, and liver **(D)**. Gene set enrichment analysis was performed using a hallmark gene set, and all data were nominal p-value < 0.05 and false discovery rate (FDR) q-value < 0.25.

Because certain kind of systemic stress may induce simultaneous GDF15 expression in various organs, we have analyzed the correlation of GDF15 expression between two different tissues. For instance, we presented the results from our correlation analyses of GDF15 expression between thyroid and other tissues (**Supplementary Figures 2A–C**). These analyses indicated neither positive nor negative correlation of thyroid with other tissues such as whole blood, muscle and liver, which means that organ specific stress might induce GDF15 expression independently from the other organ. If the expression of GDF15 is increased in multiple organs simultaneously, it would be very difficult to distinguish whether the effect of GDF15 is an autocrine effect or an endocrine effect. However, since correlation analysis did not show any association, we thought that the organ specific induction of GDF15 might be operational. Next, to investigate the effect of high GDF15 expression in whole blood, muscle and liver on thyroid gland, thyroid samples were classified into two groups according to GDF15 expression of whole blood, muscle and liver, respectively (**Figure 2A**).

We first divided the samples into quartiles based on GDF15 expression across whole blood (n=188 for each group), muscle (n=200 for each group), and liver (n=56 for each group) datasets. Next, the classified samples were matched with the thyroid samples, and samples without thyroid RNA-seq data were excluded from the classified samples. As a result, the number of thyroid samples in each group were as follows: whole blood (Low group n=76, High group n=61), muscle (Low group n=93, High group n=78), and liver (Low group n=23, High group n=31). GSEA was performed using the last classified dataset. The upregulated genes are presented in **Supplementary Tables 1–3**.

Interestingly, high GDF15 expression in blood, muscle and liver were related to similar gene set enrichment patterns of thyroid glands such as inflammatory response, TNFα signaling *via* NFκB, hypoxia, apoptosis and p53 pathway, which were also enriched gene sets by high GDF15 expression of thyroid glands itself (**Figures 2B–D**). We also identified tissue specifically enriched gene sets. High blood GDF15 expression showed coordinately enrichment of genes related to Interferon alpha response (FDR q-value=0.042, NOM p-value=0.022) and Interferon gamma response (FDR q-value=0.023, NOM p-value=0.018) genes in thyroid glands (**Figure 2B**). In the case of muscle, DNA repair (FDR q-value=0.097, NOM p-value=0.049) and Heme metabolism (FDR q-value=0.086, NOM p-value=0.016) gene set were specifically enriched in thyroid glands (**Figure 2C**). The high hepatic GDF15 expression were significantly enriched genes related to Epithelial Mesenchymal Transition (EMT) (FDR q-value=0.08, NOM p-value=0.024) and mTORC1 signaling (FDR q-value=0.09, NOM p-value=0.047) in thyroid glands (**Figure 2D**). Taken together, GDF15 expression in blood, muscle and liver might be related to the expression of specific genes of thyroid glands, which could be divided into common gene sets and organ specific gene sets.

### Liver specific GDF15 transgenic mice showed the immature morphological change of thyroid glands

**Figure 3:**
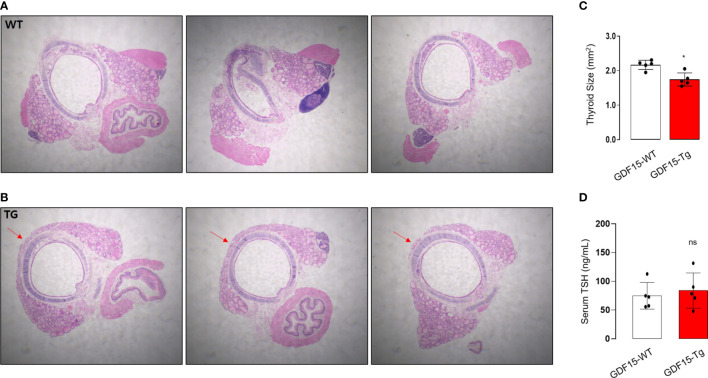
Morphological phenotype of liver-specific GDF15 transgenic (Tg) mice. **(A, B)** Representative hematoxylin and eosin staining images (40× magnification) of the thyroid glands from wild type (WT) **(A)** and GDF15-Tg mice **(B)**. Red arrows indicate the isthmus. **(C)** Comparison of thyroid gland size between the two groups (n = 5). To minimize the selection bias that the size varies depending on the location of the transverse histologic section, the largest area was measured using Image-J. **(D)** Comparison of thyroid stimulating hormone (TSH) levels between the two groups (n = 5). ^*^ Statistically significant differences in nonparametric Mann-Whitney U-test (p-value <0.05). ns, no significance.

To validate our analysis using GTEx, we observed histological phenotypes of thyroid glands from GDF15 Tg mice. Compared to control mice which have no isthmic portion, every GDF15 Tg mice showed thyroid isthmus (**Figures 3A, B**). In developmental stage, the thyroid glands in mice is divided into right and left lobes and the isthmic portion is known to eventually disappear (37). Supporting our idea, the size of thyroid glands was smaller in GDF15 Tg mice (WT = 2.19 mm2 [2.05-2.26], Tg = 1.69 mm2 [1.60-1.91], values represent medians and inter-quartile ranges) (**Figure 3C**). However, TSH in these mice was not different from control mice indicating high GDF15 expression affect the development thyroid glands independently from TSH (WT = 81.28 ng/mL [63.0-104.6], Tg = 87.57 ng/mL [66.5-123.5], values represent medians and inter-quartile ranges) (**Figure 3D**). We did not measure free T4 between GDF-15 WT and Tg mice because there was no difference in TSH levels.

**Figure 4:**
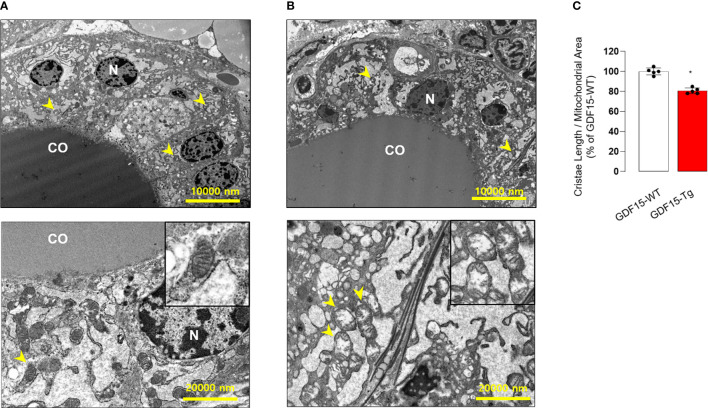
Comparison of transmission electron microscopic features between the thyroid glands from wild type (WT) and GDF15 transgenic (Tg) mice. **(A, B)** Representative images of transmission electron microscopy from WT **(A)** and GDF15 Tg **(B)** mice. High magnification images of representative mitochondria (yellow arrowheads) are shown in the insert of the corresponding panel. **(C)** Comparison of relative cristae length between the two groups (n = 5). CO: follicular lumen that contains the colloid; N: nucleus; Yellow arrowhead: mitochondria. ^*^ Statistically significant differences in nonparametric Mann-Whitney U-test (p-value <0.05).

To in depth understand the morphological changes of thyroid follicular cells, we also performed electron microscopy (TEM). Remarkably, the cristae structure of mitochondria was not well observed (**Figures 4A, B**), supported by quantitative analysis for the ratio of cristae length to mitochondria area (**Figure 4C**). In fact, differentiated endocrine cells harbors usually elongated mitochondria with well-developed cristae structure indicating active oxidative phosphorylation (38). The loss of cristae structure of thyroid glands from GDF15 Tg mice might indicate decreased oxidative phosphorylation and be another feature of immature development compatible to the presence of thyroid isthmus.

Recently, GFRAL has been known as a specific GDF15 receptor located in hindbrain (Area Postrema) as our IHC-P using hindbrain from GDF15 Tg mice confirmed GFRAL immunoreactivity in this area (**Supplementary Figure 3**). However, we could not observe any GFRAL staining signal in thyroid glands from same mice, suggesting that there was no specific GDF15 receptor (**Supplementary Figure 4**). Although we observed the morphological changes of thyroid glands in GDF15 Tg mice, this change might be induced indirectly by brain GFRAL or pleotropic effect but not be induced by direct action of GDF15 on thyroid glands.

### High serum GDF15 concentration was related to poor metabolic profile and aggressive tumor behavior in patients with PTC

Metabolic phenotype (fasting blood glucose level, fasting insulin level, HOMA-IR index, serum ALT, AST level…) in GDF15 Tg mice was reported by previous studies (28). Supporting previous findings and our data using GDF15 Tg mice, patients with high serum GDF15 had poor metabolic profiles in body mass index (BMI), systolic blood pressure (SBP), and fasting blood glucose (**Supplementary Table 4**). In addition, these patients also presented increased liver enzymes such as high alanine transaminase (ALT), alkaline phosphatase (ALP) and total bilirubin. However, these patients also did not show any difference in serum TSH level. In our correlation analysis, serum GDF15 had significant positive correlation with BMI, SBP, fasting glucose, ALT, ALP and total bilirubin (**Supplementary Table 5**). Although this data could not directly confirm that liver is the causal organ of GDF15 increase, we believed that this situation is quite similar to our data from GTEx and GDF15 Tg mice. In fact, there was some previous paper reporting poor prognosis of high GDF15 in patients with PTC (39).

**Figure 5:**
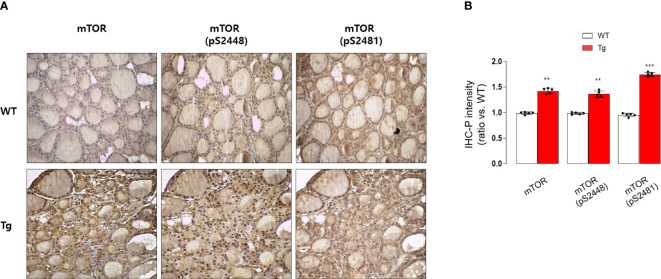
Immunohistochemical staining analysis of mTOR, pS2448 mTOR, and pS2481 mTOR in the thyroid glands from wild type (WT) and GDF15 transgenic (Tg) mice (each group n = 5). **(A)** Representative results of IHC staining (original magnification 200×). **(B)** Quantification analysis of intensities (compared with thyroid gland from wild type mice). ^**^p-value < 0.01, ^***^p-value < 0.001 (indicates a statistically significant difference from non-parametric Mann-Whitney U-test).

Consistently, our analysis for clinico-pathological features also indicated that patients with high serum GDF15 showed frequent lateral lymph node metastasis, advanced TNM stage and frequent presence of pTERT mutation (**Table 1**). Univariate and multivariate analysis clearly indicated that high serum GDF15 concentration was able to increase the risk of presence lateral lymph node metastasis and advanced TNM stage (III/IV) (**Table 2**). Using GDF15 Tg mice, we also observed increased mTOR phosphorylation and total amount of mTOR protein, verifying our GTEx result and supporting our analysis for clinico-pathological features in patients with PTC (**Figure 5**).

**Table 1:** Relationship between serum GDF15 level and clinico-pathological features of patients with PTC.

	Serum GDF15 (n = 162)		*P* value
	Low (n = 81)	High (n = 81)	
Tumor size (cm)	1.80 (1.40-2.30)	1.60 (1.35-2.25)	0.447^*^
Age (years)			
<55	70 (86.4)	63 (77.8)	0.151^†^
≥55	11 (13.6)	18 (22.2)
Multifocality			
Absent	52 (64.2)	40 (49.4)	0.121^†^
Unilateral	6 (7.4)	12 (14.8)
Bilateral	23 (28.4)	29 (35.8)
Extrathyroidal invasion			
No	26 (32.1)	24 (29.6)	0.734^†^
Yes	55 (67.9)	57 (70.4)
pT stage			
T1	21 (25.9)	15 (18.5)	0.481^†^
T2	4 (4.9)	8 (9.9)
T3	52 (64.3)	54 (66.7)
T4	4 (4.9)	4 (4.9)
Central LN^**^ metastasis			
No	22 (27.2)	26 32.1)	0.491^†^
Yes	59 (72.8)	55 (67.9)
Lateral LN metastasis			
No	60 (74.1)	45 (55.6)	0.014^†^
Yes	21 (25.9)	36 (44.4)
Distant metastasis			
M0	81 (100)	79(97.5)	0.155^†^
M1	0 (0)	2 (2.5)
pTNM stage			
I	58 (71.6)	37 (45.7)	<0.001^†^
II	0 (0)	1 (1.2)
III	19 (23.5)	22 (27.2)
IV	4 (4.9)	21 (25.9)
BRAF V600E mutation			
Absent	30 (37.0)	32 (39.5)	0.746^†^
Present	51 (63.0)	49 (60.5)
pTERT mutation			
Absent	80 (98.8)	74 (91.4)	0.030^†^
Present	1 (1.2)	7 (8.6)
Recurrence			
No	81 (100)	79 (97.5)	0.155^†^
Yes	0 (0)	2 (2.5)

GDF15, growth differentiation factor 15; PTC, papillary thyroid carcinoma; LN^**^, lymph node. ^*^ P values calculated by Mann-Whitney-U test. Data are expressed as the median and inter-quartile ranges (IQR). ^†^ P values calculated using χ^2^ test.

**Table 2:** Univariate and multivariate analyses identifying the clinico-pathological parameters that show independent correlation with serum GDF15 level.

	Univariate			Multivariate^*^		
	Odds Ratio	95% CI	*P* value	Odds Ratio	95% CI	*P* value
Age	1.818	0.80–4.14	0.155			
Gender	1.498	0.71–3.18	0.293			
Tumor size	1.042	0.78–1.40	0.781			
Multifocality	1.766	0.86–3.62	0.121			
Extrathyroidal invasion	1.111	0.57–2.18	0.759			
pT stage (III/IV)	1.126	0.57–2.21	0.731			
Central LN^**^ metastasis	1.268	0.65–2.49	0.492			
Lateral LN metastasis	3.705	1.60–8.56	0.002	3.876	1.52–9.86	0.004
pTNM stage (III/IV)	3.223	1.56–6.66	0.002	4.098	1.73–9.68	0.001
BRAF V600E mutation	1.110	0.59–2.09	0.747			
pTERT mutation	3.931	0.85–23.7	0.069			
BMI (kg/m2)	1.136	1.04–1.24	0.006	1.031	0.93–1.22	0.339
SBP (mmHg)	1.030	1.01–1.05	0.011	1.031	1.01–1.06	0.037
DBP (mmHg)	1.016	0.99–1.05	1.016			
Fasting glucose (mg/dL)	1.035	1.01–1.06	0.007	1.036	1.01–1.07	0.043
AST (IU/L)	1.012	0.98–1.04	0.426			
ALT (IU/L)	1.069	1.03–1.11	0.001	1.051	1.01–1.20	0.020
ALP (IU/L)	1.022	1.01–1.04	0.004	1.018	1.01–1.03	0.023
Total bilirubin (mg/dL)	5.519	1.58–19.32	0.008	12.616	2.06–77.35	0.006
TSH (mIU/ml)	1.331	0.93–1.91	0.118			

CI, confidence interval; LN^**^, lymph node ^*^ Adjusted for age, gender, tumor size, multifocality, and extrathyroidal invasion, central LN metastasis, Lateral LN metastasis, BMI, SBP, fasting glucose, ALT, ALP, Total bilirubin.

## Discussion

Mitochondria have an essential role in ATP production and regulate cellular adaptive response against various intracellular and extracellular stresses. These various stresses inevitably cause mitochondrial stress, promoting transactivation of key stress response transcriptional factors such ATF4, ATF5 and CHOP and secretion of mitochondrial stress-induced cytokines (mitokines) such as FGF21 and GDF15 (40). However, the cell autonomous and non-autonomous function of these novel mitokines are remained to be elucidated. GDF15 has protective role in obesity-related disease and NAFLD, whereas it showed detrimental role against age related muscle atrophy and cancer cachexia-related muscle atrophy (41–43).

Thyroid glands have crucial role in whole body energy homeostasis, which means this endocrine organ is able to determine the fate of ATP production and consumption to maintain whole body metabolic homeostasis. In fact, it is well-known that the functional abnormality such as hyperthyroidism and hypothyroidism is related to metabolic abnormality. However, the effect of metabolic disease on thyroid glands are not fully investigated. In this study, first we aimed to investigate the effect of GDF15 from metabolically active organs on thyroid glands using GTEx and interestingly found that high *GDF15* expression in in blood, muscle and liver were related to the coordinately upregulation of gene sets of thyroid glands. Performing GSEA, when *GDF15* was high in muscle, liver, and blood, the gene sets whose expression was increased in the thyroid gland could be divided into two sets; common gene sets and organ specific gene sets. Most of common gene sets were associated with inflammatory response and cellular apoptosis. The high hepatic *GDF15* expression was linked to EMT and mTORC1 signaling with organ specific manner. Based on this observation, we moved to validate our GTEx analysis using *GDF15*-Tg mice, where we observed the two lobes connected by an isthmus portion overlying upper trachea. By contrast, we could not find any isthmus portion, resulting in separate two lobes in control mice. However, it appears that the different shape of thyroid glands has little functional effect, because our laboratory test for TSH was not different. Indeed, athyroid mice generated by high dose of radioactive iodine could be rescued by implanting thyroid follicles from mouse embryonic stem cells (ESC) into the kidney (44). These results have suggested that the maintenance of the follicular structure rather than the shape of the organ plays an important role in the production of thyroid hormone. But, the small-sized thyroid gland and the presence of isthmus might have an impact on the risk or natural course of other diseases such as tumors although this speculation is very presumptive. This may be helpful in understanding the risk and course of diseases occurring in the thyroid gland when mitochondrial stress is generated by in the other organs.

Remarkably, we found oncogenic signaling such as EMT and mTORC1 signaling was upregulated in thyroid glands from high liver GDF15 human using GTEx data. These tumor-related signals are known to inhibit mitochondrial function, so called Warburg’s effect. Supporting this hypothesis, our EM findings indicated loss of mitochondrial cristae, which means depressed mitochondrial oxidative phosphorylation in GDF15-Tg mice. Based on this findings, we thought that high liver GDF15 expression might affect the tumor development and/or progression in thyroid glands.

In fact, mitochondrial retrograde signaling, a new area of mitochondrial research, have been focusing on the pathways by which dysfunctional mitochondria communicate with nuclear genetic compartments to convey prevailing metabolic, oxidative, and respiratory states in mitochondria (45, 46). Through this approach, the cross-talk between mitochondrial retrograde signaling pathway and critical stress response including rapamycin target (TOR) has been investigated (47). Compatible to these previous reports, activation of the mitochondrial retrograde signaling pathway in cancer cells affects the upregulation of genes that affect apoptosis resistance, multidrug resistance, invasion, and several cellular functions including EMT (48, 49). As the previous study, this intracellular retrograde signaling could generate poor prognostic features of PTC (39). Interestingly, our study suggested that mitochondrial retrograde signaling is not limited to intracellular signals, but it can also cause nuclear reprogramming in other organs simultaneously. In line with this idea, our analysis also presented aggressive tumor behavior of PTC in patients with high serum GDF15 concentration.

In fact, circulating GDF15 stimulate Area Postrema (AP) neuron *via* GFRAL located exclusively in the nucleus of the tractus solitarius (NTS) and AP regions of the brain stem in mice (25–27, 50, 51) as our IHC-P using commercially available antibody. Compatible to these previous reports, we could not find any signal intensity of GFRAL in mouse thyroid, indicating liver GDF15 might act on thyroid glands *via* hind brain GFRAL and/or indirectly *via* pleiotropic effect. Unfortunately, in this paper, we could not define which mechanism affects the thyroid gland. However, the effect of GDF15 on thyroid glands appears to be independent of TSH and is expected to be an important data in clarifying various factors affecting the growth and development of the thyroid including insulin and transforming growth factor-β (TGF-β) (52–54).

Our clinical data did not directly prove a causal relationship for hepatic GDF15 in promoting the aggressiveness of PTC and did not demonstrate mTOR signaling activation in the high GDF15 group. However, our data suggested a possibility that serum GDF15 might originate from both PTC and diseased metabolic organs. This cell non-autonomous crosstalk between cancer and metabolic organs would be a good topic for future research.

In summary, increased expression of hepatic GDF15 is involved in the development of the thyroid gland by forming the thyroid isthmus by mechanisms independent of the serum TSH level and GDF15 receptors GFRAL, suggesting the presence of cell non-autonomous regulatory mechanism form liver to thyroid glands affecting thyroid cancer behavior.

## Data availability statement

The original contributions presented in the study are included in the article/**Supplementary Material**. Further inquiries can be directed to the corresponding authors.

## Ethics statement

The studies involving human participants were reviewed and approved by Institutional Review Board of Severance Medical Center (Seoul, South Korea) on 30 September 2013 (No. 4-2013-0546). The patients/participants provided their written informed consent to participate in this study. The animal study was reviewed and approved by Institutional Animal Care and Use Committee of Yonsei University Health System.

## Author contributions

JL and YJ designed the project and supervised the research. SJ, SL, KK, WL, XZ and SP performed the experiments and analyses. HL, SL, WL collected and provided patients samples and clinical data. SJ and SL performed the computational analysis. M-SL and S-YC contributed to discussions about the research. SJ, SL, JL, and YJ wrote the manuscript; all authors reviewed the manuscript. All authors contributed to the article and approved the submitted version.

## Funding

SL was supported by National Research Foundation of Korea (NRF) grants funded by the Korean government (MEST) (NRF-2020R1F1A1048986). YJ was supported by National Research Foundation of Korea (NRF) grants funded by the Korean government (MEST) (NRF-2018R1A2B6004179). JL was supported by a National Research Foundation of Korea (NRF) grant funded by the Korea government (MEST) (NRF-2020R1A2C1006047) and by the Korean Foundation for Cancer Research (2020), and by the faculty research grant of Yonsei University College of Medicine (6–2020–0085).

## Acknowledgments

The authors would like to thank Ji Young Kim (Severance Hospital), Hee Chang Yu (Severance Hospital), and Ho young Kim (Severance Hospital) for technical support. And We also would like to thank Editage (www.editage.co.kr) for English language editing.

## Conflict of interest

The authors declare that the research was conducted in the absence of any commercial or financial relationships that could be construed as a potential conflict of interest.

## Publisher’s note

All claims expressed in this article are solely those of the authors and do not necessarily represent those of their affiliated organizations, or those of the publisher, the editors and the reviewers. Any product that may be evaluated in this article, or claim that may be made by its manufacturer, is not guaranteed or endorsed by the publisher.
