# Supplementary material for: Cell non-autonomous effect of hepatic growth differentiation factor 15 on the thyroid gland
Source: Front Endocrinol (Lausanne). 2022 Aug 15;13:966644. doi: 10.3389/fendo.2022.966644 (PMC9420875; doi:10.3389/fendo.2022.966644)
Supplement: Supplementary file 1 [file DataSheet_1.pdf]

Supplementary Material

1 Supplementary Figures

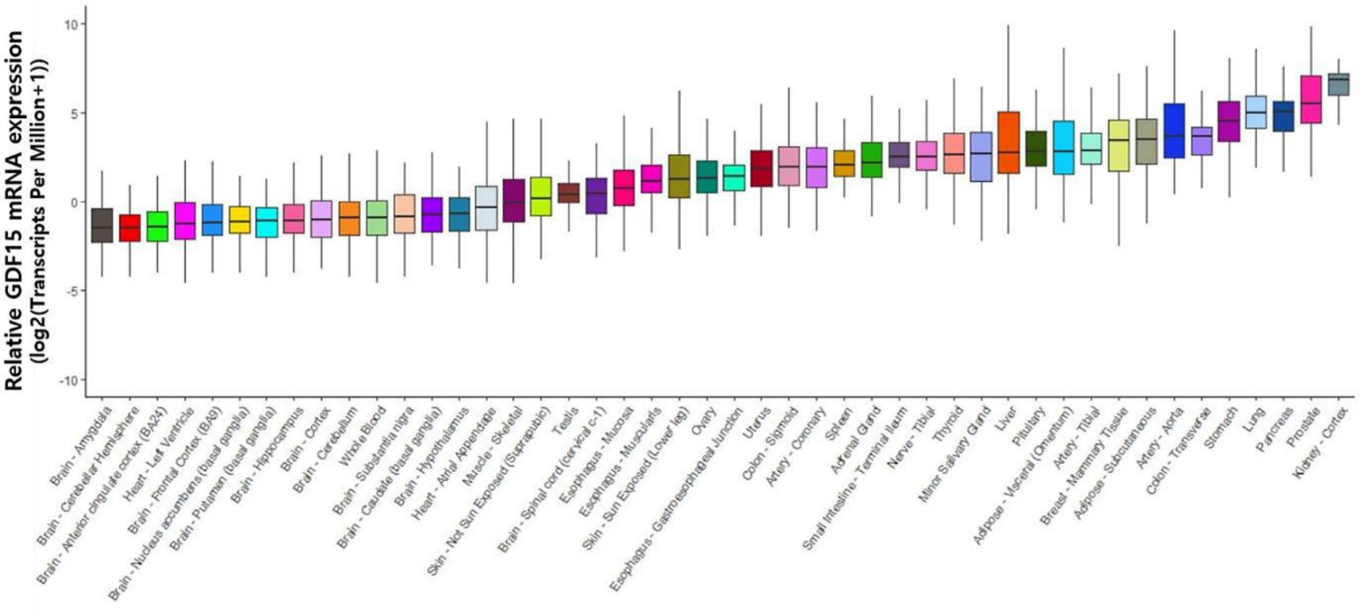

Supplementary Figure 1. Comparison of GDF15 expression in human tissues. GDF15 mRNA expression was analyzed in 46 types of human tissues using Genotype-Tissue Expression (GTEx) portal data. The horizontal solid bars of each box represent the median; the upper and lower bounds of the box represent the third and first quartiles of the data, respectively; and the whisker bars above and below the box are the 90th and 10th percentiles, respectively.

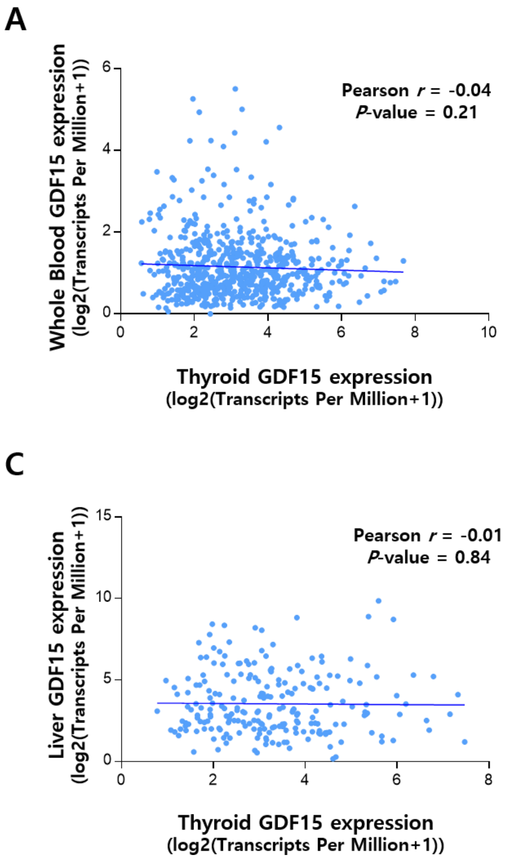

Supplementary Figure 2. Correlation analysis of thyroid GDF15 expression with whole blood, muscles, and liver GDF15 expression. Correlation of thyroid GDF15 expression (n = 653) with GDF15 expression in **(A)** whole blood (n = 755), **(B)** muscles (n = 802), and **(C)** liver (n = 226). Correlation analysis was performed using Pearson's correlation coefficient.

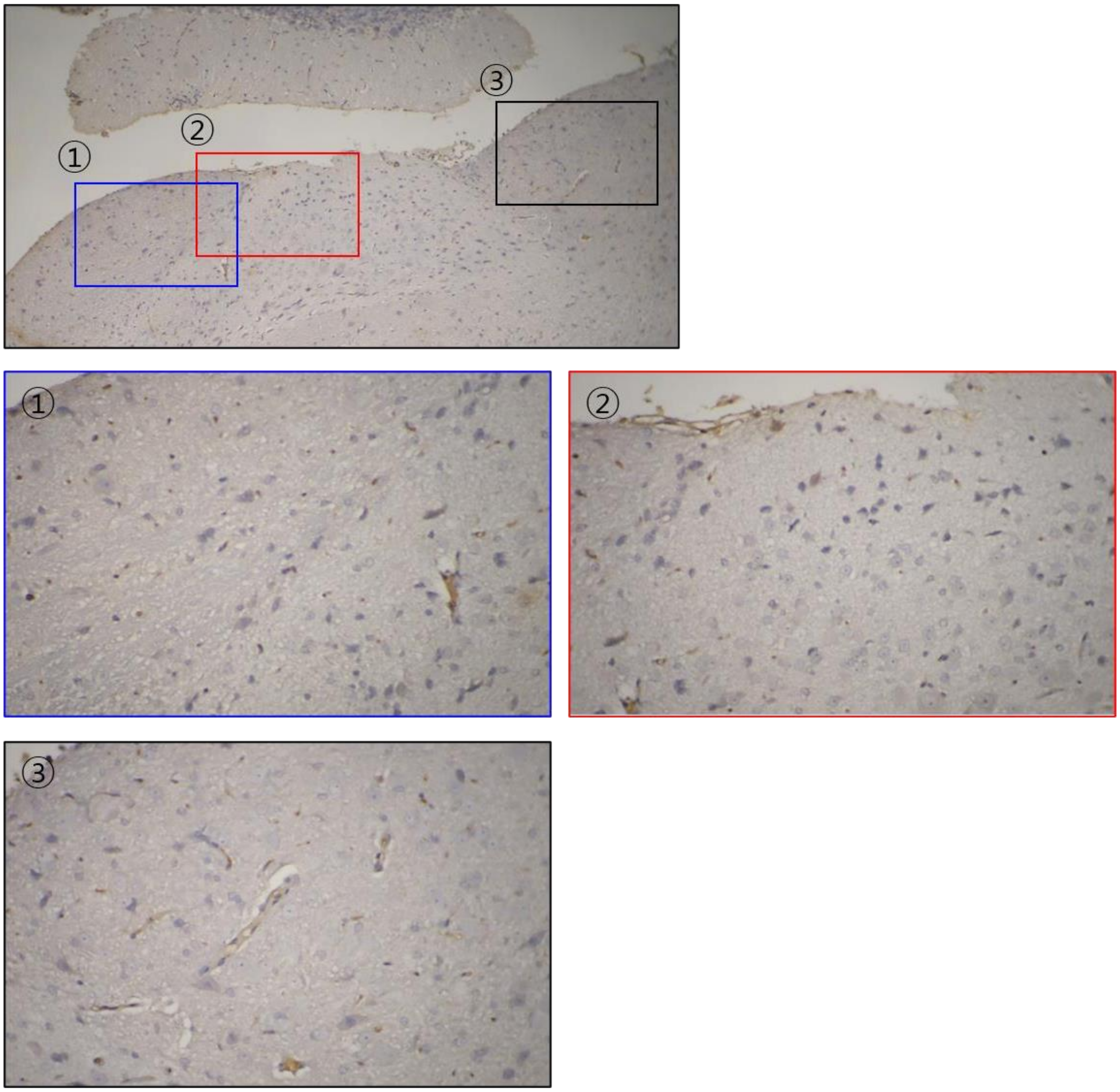

Supplementary Figure 3. Representative images of GFRAL immunohistochemistry-paraffin (IHC-P) staining in the hindbrain (area postrema) region of GDF15 Tg mice.

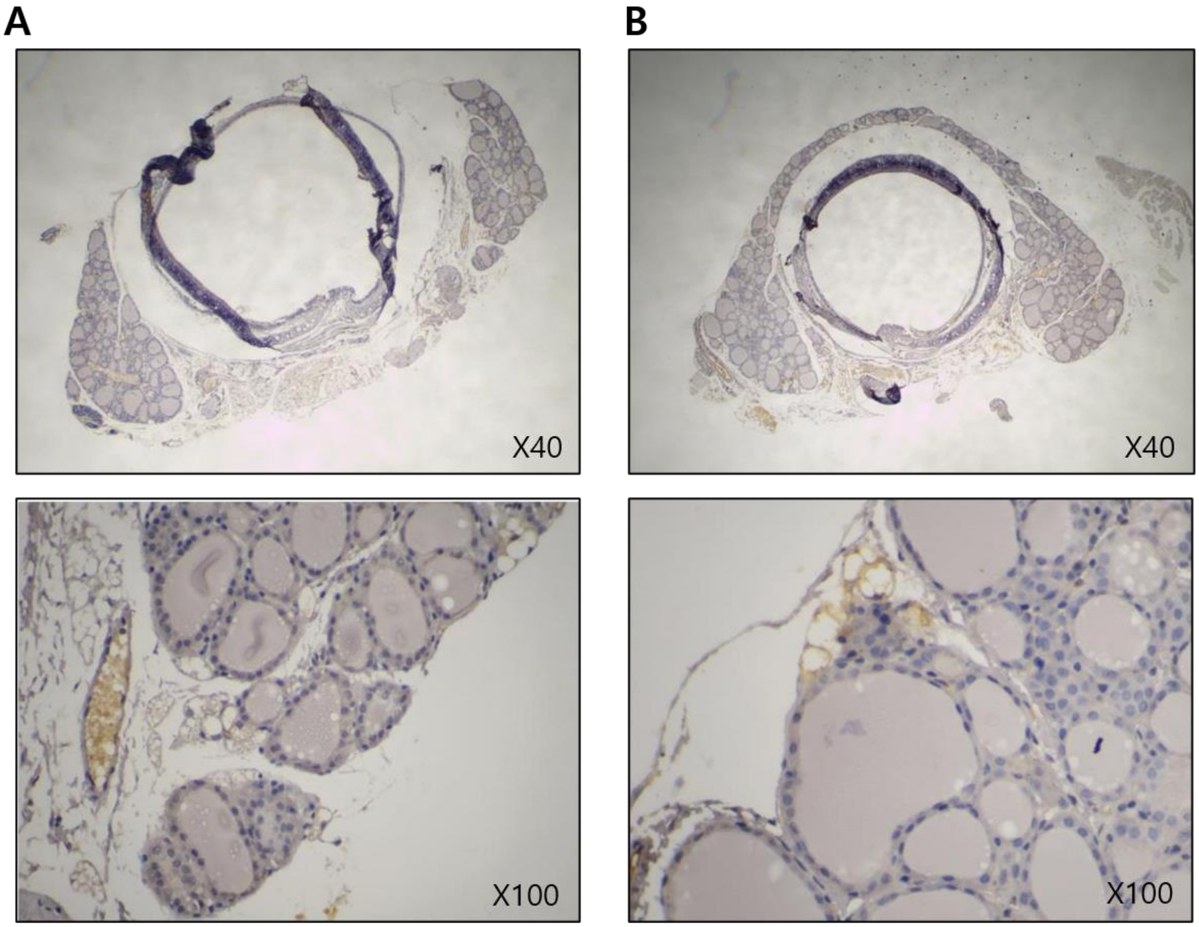

Supplementary Figure 4. Representative images of GFRAL immunohistochemistry-paraffin (IHC-P) staining of the thyroid glands from (A) wild type and (B) GDF15 Tg mice.

2 Supplementary Tables

Supplementary table 1. Genes upregulated in whole blood and thyroid group with high GDF15 expression.

Geneset	-log10(FDR-*q*val)	Gene lists
P53 PATHWAY	5.00	BLCAP,HSPA4L,PVT1,RAD51C,NUDT15,CDKN2AIP,BMP2,IP6K2,COQ8A,TAX1BP3,CTSD,IER5,PERP,GLS2,ITGB4,CDKN2A,NOL8,TRIB3,RPS12,NUPR1,SERTAD3,F2R,TGFB1,FDXR,CTSF,TAP1,DDB2,BTG1,RAD9A,HRAS,PITPNC1,PTPRE,CCNG1,ALOX15B,CCNK,TM4SF1,GADD45A,PHLDA3,LIF,NOTCH1,CASP1,MKNK2,ERCC5,CD81,RPS27L,RPL36,ADA,RRA D,FOS,NINJ1,ST14,BTG2,TSC22D1,ISCU,S100A10,RAB40C,STOM,RACK1,PRMT2,CDKN2B,ZMAT3,PDGFA,RPL18,PIDD1,SAT1,RGS16,SPHK1,VWA5A,RNF19B,PLXNB2,MXD1,DDIT3,NDRG1,IER3,HBEGF,ATF3,PLK2,WRAP73,JUN,TNFSF9,UPP1,INHBB,CDKN1A,PLK3,ZFP36L1,RALGDS,FAS,PPP1R15A
TNFA SIGNALING VIA NFKB	4.00	CSF2,ZBTB10,BMP2,DENND5A,JAG1,DUSP2,KLF10,IER5,EDN1,NR4A3,NFKB1,EGR2,PHLDA2,IER2,TNF,PANX1,SPSB1,SERPINB2,SMAD3,FOSL1,IL6,PMEPA1,SDC4,BCL6,TAP1,B TG1,TNFAIP6,LAMB3,SLC16A6,PTPRE,REL,KLF6,CEBPB,LITAF,GADD45A,NAMPT,IL15RA,LIF,CCL20,B4GALT5,RCAN1,NFE2L2,BIRC2,TRAF1,GCH1,MARCKS,NR4A2,CCRL2,EGR1, FJX1,MCL1,BCL2A1,BTG3,TANK,NFIL3,G0S2,RIPK2,EGR3,FOS,NINJ1,IRF1,PLAU,BTG2,CEBPD,GADD45B,NFKBIE,GFPT2,TSC22D1,CSF1,OLR1,ACKR3,RELA,DNAJB4,TNFAIP2,CD8 3,TNFAIP3,CCNL1,CXCL1,TNIP1,SOD2,EIF1,TGIF1,NR4A1,MAP3K8,ZC3H12A,FOSL2,MAP2K3,ETS2,PTGER4,SOCS3,CXCL3,SERPINE1,SAT1,CXCL2,PDE4B,SPHK1,CD44,IFNGR2,I CAM1,RNF19B,MXD1,RELB,CCL2,IER3,EFNA1,HBEGF,ATF3,KDM6B,PNRC1,TNIP2,TRIP10,PLK2,DUSP5,MSC,JUN,TNFSF9,JUNB,BCL3,CDKN1A,MAFF,PLAUR,PFKFB3,PTGS2,ZFP36,NFKB2,GEM,SLC2A3,BHLHE40,PPP1R15A
IL6 JAK STAT3 SIGNALING	2.17	IL17RB,TNFRSF12A,STAT1,IL1B,A2M,PF4,IL7,IL9R,DNTT,CSF2,STAT3,CRLF2,HAX1,IL18R1,CBL,TNF,CSF2RB,IL13RA1,TGFB1,IL6,STAT2,INHBE,PIM1,CD9,TNFRSF1B,OSMR,IL15RA,IRF9,TNFRSF21,IL1R1,IFNAR1,TNFRSF1A,IRF1,TYK2,IL10RB,CSF3R,CSF1,LTBR,CXCL1,MAP3K8,SOCS3,CXCL3,PTPN2,CD44,IFNGR2,IL4R,IL3RA,LEPR,JUN,FAS
IL2 STAT5 SIGNALING	2.14	PHLDA1,GLIPR2,SPP1,PLEC,GBP4,CDKN1C,IRF6,DCPS,GALM,CISH,HIPK2,RNH1,CSF2,BMP2,DENND5A,SERPINB6,ADAM19,SMPDL3A,PUS1,IL18R1,MUC1,ITGAE,MYO1E,P2RX4,TNFSF10,MAP6,SPRED2,DRC1,IL1RL1,IFITM3,ITGAV,PIM1,P4HA1,AHR,PRNP,TNFRSF1B,KLF6,COL6A1,TNFRSF4,PENK,WLS,RRAGD,LIF,SOCS2,TRAF1,TNFRSF21,FGL2,CD81,P NP,SELP,UCK2,GABARAPL1,NRP1,NFIL3,SYNGR2,GADD45B,LRIG1,CSF1,SCN9A,IKZF4,SNX9,GATA1,CD83,NFKBIZ,CA2,PLAGL1,MAP3K8,FLT3LG,ECM1,CAPN3,RGS16,GPX4,C D44,BATF3,MXD1,PLSCR1,NDRG1,IL4R,IL3RA,ST3GAL4,MAFF,SLC2A3,BHLHE40
INFLAMMATORY RESPONSE	1.98	SRI,APLNR,IL15,MET,SELE,EDN1,IL18R1,NFKB1,GPC3,STAB1,P2RX4,GPR132,TNFSF10,NMI,ITGB8,CSF3,C5AR1,IL6,CX3CL1,PDPN,IL18RAP,TNFAIP6,AHR,MEFV,TNFRSF1B,PTPR E,LAMP3,KLF6,OSMR,TNFSF15,HPN,NAMPT,IL15RA,LIF,ABI1,HIF1A,CCL20,GCH1,TIMP1,IL1R1,IFNAR1,CD40,CCRL2,HRH1,LY6E,BST2,RIPK2,IRF1,BTG2,IRF7,CSF3R,CSF1,LYN,B EST1,IFITM1,CXCL8,OLR1,PROK2,IRAK2,ITGA5,RELA,CD55,TPBG,PVR,PTGER4,GABBR1,SERPINE1,PDE4B,RGS16,SPHK1,IFNGR2,ICAM1,MXD1,CCL2,PTGIR,HBEGF,IL4R,TAPBP,OSM,TNFSF9,ADM,CDKN1A,PLAUR
APOPTOSIS	1.85	CASP6,FASLG,CFLAR,IFNB1,DCN,EREG,BAX,MMP2,SOD1,ERBB2,NEDD9,CD69,RNASEL,DFFA,TNFRSF12A,IL1B,PPP3R1,WEE1,FEZ1,CREBBP,DPYD,SATB1,ERBB3,BIRC3,CLU,BMP2,TGFB2,PMAIP1,SMAD7,CDC25B,LMNA,BID,MGMT,DNAJA1,TNF,GPX1,TNFSF10,BCL2L11,TSPO,CDK2,F2R,IL6,FDXR,IFITM3,TAP1,PLAT,SLC20A1,GADD45A,SPTAN1,HSPB1, ANXA1,PEA15,BGN,CASP1,GCH1,LUM,CASP7,TIMP1,MCL1,CASP4,BTG3,EGR3,IRF1,BTG2,GADD45B,GUCY2D,TIMP2,HMGB2,AIFM3,BCL10,RELA,PPP2R5B,ANKH,SOD2,RHOT2,ENO2,RARA,LGALS3,SAT1,CDKN1B,GPX4,CD44,HGF,CASP9,DDIT3,IER3,ATF3,JUN,CDKN1A,FAS
HYPOXIA	1.82	SLC2A5,GCNT2,IGFBP3,MAP3K1,HDLBP,NAGK,DUSP1,LDHA,NDST1,ISG20,WSB1,HSPA5,EFNA3,SDC3,CASP6,SLC25A1,STBD1,GPC4,DCN,KDELR3,PKLR,LARGE1,PFKP,GCK,GPC1,HAS1,CA12,SULT2B1,ETS1,B4GALNT2,AMPD3,CDKN1C,KDM3A,TPST2,PDGFB,NDST2,ILVBL,PGM2,COL5A1,P4HA2,GPC3,NR3C1,PPFIA4,SIAH2,ANGPTL4,ERRFI1,AKAP12,T PD52,INHA,NOCT,EXT1,IL6,SDC4,PFKL,AK4,PIM1,RBPJ,BTG1,P4HA1,ANKZF1,MYH9,PAM,CITED2,KLF6,RRAGD,TGFBI,BGN,MXI1,PHKG1,CCNG2,GAA,HEXA,EDN2,LXN,NFIL3,F OS,SCARB1,JMJD6,ACKR3,TKTL1,TNFAIP3,TGFB3,IDS,TPBG,FOSL2,ENO2,SERPINE1,CDKN1B,ERO1A,CP,DDIT3,NDRG1,IER3,EFNA1,ATF3,PNRC1,JUN,STC2,ADM,CDKN1A,MAF F,PLAUR,STC1,PFKFB3,ZFP36,SLC2A3,BHLHE40,PPP1R15A
INTERFERON GAMMA RESPONSE	1.65	C1R,PARP12,CMTR1,EIF4E3,CD69,DDX60,PFKP,C1S,CIITA,HERC6,STAT1,LAP3,HLA_G,MTHFD2,IFIT1,OAS2,GBP4,IL7,CFH,PSMB9,DDX58,STAT4,SRI,CFB,CD74,RNF213,STAT3,IL15,HLA_DRB1,SSPN,PSMB8,IFI27,OAS3,NFKB1,MX1,IFI35,SERPING1,OASL,DHX58,PARP14,CSF2RB,TNFSF10,NMI,PSME1,TRIM25,HLA_A,MX2,ISG15,IL6,STAT2,APOL6,IFI44L,HLA_DMA,IFITM3,TAP1,ARID5B,PIM1,BTG1,IFI44,UBE2L6,HLA_B,ISOC1,TNFAIP6,LGALS3BP,CD274,IRF2,NAMPT,IL15RA,IRF9,B2M,HIF1A,IL18BP,IFITM2,CASP1,FGL2,GCH1,IFNAR2,PNP,SELP,CASP7,CD40,ST8SIA4,CASP4,LY6E,BST2,XAF1,RIPK2,IRF1,SP110,PSME2,IRF7,LYSMD2,SECTM1,TNFAIP2,TNFAIP3,SOD2,MVP,NOD1,SOCS3,OGFR,PDE4B,PTPN2,S LC25A28,HELZ2,ICAM1,VCAM1,CCL2,PLSCR1,IL4R,TAPBP,AUTS2,PML,PELI1,UPP1,CDKN1A,RBCK1,PTGS2,FAS
KRAS SIGNALING UP	1.63	EREG,GPRC5B,RELN,HSD11B1,MYCN,PRDM1,CPE,PPBP,ADGRA2,ST6GAL1,USH1C,IL1B,MMP10,ENG,MMP9,CBX8,ETS1,INHBA,PLVAP,TMEM176B,EPB41L3,SPP1,TRIB2,AVL9,S ATB1,IGF2,BIRC3,ACE,CFH,TSPAN13,STRN,CFB,ADGRL4,CSF2,GPNMB,BMP2,TMEM158,WDR33,ITGBL1,LY96,PSMB8,FGF9,CBL,TSPAN7,TMEM176A,HOXD11,PRKG2,ANGPTL4,RETN,GNG11,AKAP12,TMEM100,AKT2,JUP,CCSER2,BTC,ITGA2,TNFRSF1B,PLAT,GYPC,HDAC9,LIF,CCL20,PRRX1,TRAF1,ZNF277,NRP1,G0S2,PLAU,GFPT2,SDCCAG8,MMP11,MPZL2,ALDH1A3,ADAM8,IL33,TNFAIP3,CA2,ANO1,ANKH,TSPAN1,FLT4,DUSP6,IL1RL2,USP12,SEMA3B,RGS16,VWA5A,ERO1A,HBEGF,PLAUR,PTGS2,PPP1R15A
INTERFERON ALPHA RESPONSE	1.37	EPSTI1,RSAD2,ISG20,EIF2AK2,IFIT3,SAMD9L,PNPT1,PARP12,CMTR1,DDX60,C1S,HERC6,PROCR,GMPR,LAP3,GBP4,IL7,OAS1,PSMB9,CD74,HLA_C,IL15,PSMB8,IFI27,CD47,MX1, IFI35,OASL,DHX58,PARP14,NMI,PSME1,TRIM25,ISG15,STAT2,IFI44L,IFITM3,TAP1,IFI44,UBE2L6,LGALS3BP,LAMP3,PARP9,TMEM140,IRF2,IRF9,B2M,IFITM2,CASP1,MVB12A,CC RL2,LY6E,BST2,RIPK2,IRF1,SP110,UBA7,PSME2,IRF7,CSF1,IFITM1,MOV10,GBP2,OGFR,LPAR6,SLC25A28,HELZ2,NCOA7,PLSCR1,IL4R

Supplementary table 2. Genes upregulated in muscle and thyroid group with high GDF15 expression.

Geneset	-log10(FDR-*q*val)	Gene lists
P53 PATHWAY	2.04	TGFB1,KRT17,BAX,SERTAD3,CTSD,SESN1,LIF,TRIB3,EI24,HSPA4L,SFN,CCNK,ADA,SAT1,GADD45A,PLK3,TXNIP,NUDT15,XPC,CGRRF1,F2R,NUPR1,RRP8,POM121,RCHY1,CD8 1,HINT1, PVT1,TSC22D1,ZNF365,FDXR,CCND2,TRIAP1,TAP1,CSRNP2,PHLDA3,JUN,RGS16,ITGB4,RRAD,CDKN2AIP,PITPNC1,S100A10,ST14,RAB40C,HBEGF,SPHK1,IP6K2,HR AS,SLC35D1,PPM1D, CDKN1A,ABCC5,ATF3,IER3,TPRKB,STOM,WWP1,APAF1,NINJ1,CYFIP2,RAD51C,NDRG1,ISCU,COQ8A,PERP,BTG1,MKNK2,RPS27L,ERCC5,UPP1,TM4SF1, CCP110,PIDD1,RAD9A,CASP1, PDGFA,PRMT2,PLK2,SP1,RALGDS,NOTCH1,DDB2,ZMAT3,PPP1R15A,FAS,CTSF,WRAP73,DDIT3,ZFP36L1,RPS12,FGF13,RNF19B,RACK1,RPL36,MXD1,RPL18,GLS2,PLXNB2,VWA5A,NOL8, CCNG1,INHBB
HEME METABOLISM	1.06	LPIN2,VEZF1,TYR,ANK1,HAGH,ISCA1,ASNS,BNIP3L,DAAM1,ARHGEF12,MPP1,KDM7A,LMO2,UBAC1,HEBP1,BPGM,EPB41,USP15,KHNYN,SLC6A9,RHD,GYPB,GYPA,SPTA1,TC EA1, CIR1,SEC14L1,P4HA2,DMTN,HDGF,ALAD,LRP10,ACKR1,CA2,FOXJ2,OSBP2,PGLS,MAP2K3,DCUN1D1,EPB42,MOCOS,MINPP1,FTCD,CDR2,GYPC,ADIPOR1,NR3C1,CTNS, BTRC,SPTB, RBM38,SMOX,SDCBP,MKRN1,SNCA,FBXO34,AHSP,TRIM10,CROCCP2,MBOAT2,FBXO7,HBQ1,CAST,CA1,BCAM,MFHAS1,YPEL5,CLIC2,GATA1,NFE2,CCDC28A,HB D,ALAS2,KLF1, SLC4A1,PPOX,RBM5,TRAK2,NEK7,IGSF3,OPTN,HTRA2,MOSPD1,MXI1,HBB,CPOX,NARF,RANBP10,EZH1,SIDT2,EPOR,PPP2R5B,TMCC2,SLC25A37
INFLAMMATORY RESPONSE	1.06	C5AR1,ICOSLG,GCH1,BEST1,STAB1,P2RY2,TNFAIP6,DCBLD2,PTGER4,KCNMB2,LIF,LY6E,MMP14,NAMPT,IRF1,CXCL6,CCL2,IL18RAP,CCL20,IL18R1,ICAM1,CD55,SLC28A2,AP LNR,SRI, IL6,AQP9,PDE4B,ITGB8,IL15RA,OPRK1,HIF1A,MET,EIF2AK2,TNFSF15,ITGA5,NFKB1,ADM,OLR1,TACR1,SCN1B,LYN,LAMP3,ABI1,IL15,HPN,P2RX7,ATP2C1,PDPN,RGS1 6,TIMP1,CCRL2, P2RX4,TNFRSF1B,VIP,CSF3R,HBEGF,PVR,SPHK1,CDKN1A,CXCL8,AHR,OSM,IL1R1,IRF7,NMI,TNFSF10,PROK2,BST2,IRAK2,CD40,MEFV,HRH1,IFNAR1,CSF1,IL4R,FZD5,OSMR,IFITM1, RIPK2,PLAUR,RELA,IFNGR2,PTGIR,MXD1,TAPBP,GABBR1,TPBG,KCNJ2
DNA REPAIR	1.01	VPS28,RNMT,POLR2K,RAE1,POLR2D,TAF10,MPC2,NCBP2,TYMS,SSRP1,SAC3D1,POLR3GL,DGUOK,PRIM1,REV3L,ADA,NUDT9,XPC,ERCC8,SNAPC5,PDE4B,ELL,POLR2I,POLA1,POM121, GTF2A2,MPG,HPRT1,TK2,ERCC1,GTF2F1,STX3,ZNF707,POLR2F,SUPT4H1,TAF1C,POLD4,NELFE,NME4,ERCC2,CSTF3,SUPT5H,TAF6,AGO4,NUDT21,NELFB,SDCBP,POLR2J,GMPR2,BCAM, SRSF6,RFC2,CANT1,ERCC5,AAAS,GUK1,NPR2,EIF1B,ERCC3,SNAPC4,VPS37D,NT5C3A,DDB2,RALA,ZNRD1,NME3,SMAD5,GPX4,POLR2H,POLE4,POLD1,POLD3,DUT,GTF3C5,RAD52,RFC4,IMPD H2,LIG1,ADCY6,TAF12,POLL,POLB,POLR1D,UPF3B,AK3
APOPTOSIS	1.00	LEF1,DAP3,EGR3,GCH1,BNIP3L,BAX,WEE1,SOD1,ERBB2,MGMT,MADD,IRF1,ANXA1,CCNA1,SAT1,GADD45A,TXNIP,TSPO,IL6,FEZ1,F2R,BIRC3,BCL2L2,SLC20A1,RARA,IFITM3,FDXR, PLAT,CCND2,TAP1,SOD2,MCL1,LUM,BGN,JUN,CASP6,BTG3,TIMP1,PPP3R1,TIMP2,RHOT2,HSPB1,BCL2L11,DNAJA1,BMF,SMAD7,CDK2,DPYD,AIFM3,CDKN1A,ATF3,D FFA,IER3,BID, TNFSF10,LGALS3,CDC25B,CASP9,CASP1,ANKH,BCL10,CASP4,PEA15,RELA,GPX4,FAS,HMGB2,GUCY2D,SATB1,ENO2,DDIT3,ERBB3,CDKN1B,PPP2R5B,CASP7,RNASEL,HGF,CD44
KRAS SIGNALING UP	0.98	MMD,DNMBP,ETV1,MMP10,ADGRA2,CSF2,MAP3K1,MMP9,ARG1,TFPI,APOD,NR1H4,ANGPTL4,CBR4,CBX8,PLVAP,PDCD1LG2,CPE,TSPAN1,MTMR10,RETN,PLEK2,SNAP25,GPNMB, GPRC5B,DCBLD2,SCG5,ETS1,PECAM1,LIF,MYCN,ITGBL1,EVI5,BPGM,G0S2,CCL20,CFH,ALDH1A2,HDAC9,TRIB2,ENG,SPP1,TMEM100,BIRC3,PTCD2,FLT4,TMEM158,S T6GAL1,TNFAIP3, TOR1AIP2,PLAT,TSPAN13,NIN,CCND2,CA2,ALDH1A3,SCN1B,RBM4,EPB41L3,AVL9,RGS16,PSMB8,GFPT2,GYPC,ADGRL4,CFB,TNFRSF1B,PRRX1,HBEGF,SEM A3B,GNG11,ADAM8, TMEM176B,PTGS2,CBL,PLAU,SDCCAG8,STRN,USP12,WDR33,CCSER2,ANO1,FGF9,NRP1,PPBP,ANKH,MMP11,MPZL2,PLAUR,PPP1R15A,ITGA2,TMEM176A,LY96,ERO1A,TRAF1,SATB1,BTC,IL1R L2,ZNF277,DUSP6,VWA5A,IL33
MITOTIC SPINDLE	0.96	FSCN1,CDC42BPA,CENPF,CENPE,LATS1,KNTC1,CLIP1,ANLN,ARHGEF12,TAOK2,ARF6,PLEKHG2,ARL8A,FGD6,RASA1,BCR,KIFAP3,ITSN1,SAC3D1,TUBGCP6,RFC1,EPB41,CEP5 7, DYNLL2,MID1IP1,MYO1E,ARFGEF1,RACGAP1,KIF2C,CKAP5,CNTRL,TLK1,EPB41L2,NCK2,BIN1,MAP3K11,MYO9B,ECT2,NIN,KIF15,PXN,NOTCH2,ARHGEF3,NUMA1,ABI1,CD2AP, DST,BCAR1,WASL,HDAC6,CEP192,TSC1,RHOT2,PKD2,BCL2L11,RAPGEF5,FBXO5,CDC42EP2,KIF3B,MYH9,HOOK3,ARHGAP5,ARAP3,PLK1,PPP4R2,TUBGCP2,FLNB,LMNB1,ALMS1, RANBP9,KLC1,DOCK4,ARHGEF7,KIF5B,LLGL1,OPHN1,MARK4,SASS6,RALBP1,WASF1,SYNPO,TBCD,CEP250,ALS2,PCGF5,NCK1,CTTN,CDK5RAP2,CEP131,CNTROB,GEMIN4,UXT, SHROOM2,KPTN,MID1,CLIP2,ARHGEF2,ATG4B,KIF3C,CDC42EP1,FARP1,MARCKS,CYTH2,SHROOM1,SOS1,RASA2,TUBD1,PCM1
APICAL JUNCTION	0.91	ACTN2,ITGB1,ACTC1,NRAP,COL9A1,VCAN,MMP9,MSN,CDH6,SDC3,CDSN,KCNH2,CNTN1,CRB3,NLGN3,ITGA9,CDH15,RRAS,CDH8,FSCN1,SKAP2,MPP5,FLNC,EXOC4,CD99,ZYX, TAOK2,MPZL1,ADAM23,NECTIN4,RASA1,PECAM1,CNN2,AMIGO1,CLDN6,ACTG1,SRC,ARHGEF6,ICAM2,CADM2,ICAM1,ACTN1,YWHAH,PARVA,CD209,LIMA1,VAV2,AD AM9,ITGA3, EPB41L2,VWF,GTF2F1,CD276,SORBS3,TSPAN4,CLDN9,GNAI2,MAPK14,ITGB4,WASL,LAMB3,TSC1,AMH,LDLRAP1,TMEM8B,LAYN,PBX2,DHX16,MYH9,COL16A1, MAP3K20,INPPL1,CDH3, CDK8,HRAS,CLDN5,CERCAM,CDH11,GAMT,MAPK11,NRXN2,CRAT,TRO,PIK3CB,NFASC,TGFBI,AKT3,SGCE,ICAM5,JAM3,MDK,CD274,SIRPA,TIAL1,PKD1,BMP1,MPZL2,SHROOM2, VCAM1,EVL,ITGA2,CLDN15,TRAF1,CLDN4,STX4,GRB7,MAPK13,CLDN18,SYMPK,THBS3,NLGN2
TNFA SIGNALING VIA NFKB	0.81	IL6ST,JAG1,KLF6,FOS,TNFAIP8,CSF2,NR4A2,DRAM1,CFLAR,ACKR3,IER5,TNFSF9,GADD45B,DDX58,IER2,EDN1,SDC4,NR4A1,EGR3,SLC2A6,SNN,MAP3K8,ICOSLG,GCH1,SLC16A6, TNFAIP6,STAT5A,PTGER4,LIF,EGR1,CXCL3,NAMPT,REL,KLF10,IRF1,CXCL6,G0S2,CCL2,CCL20,IFIH1,SAT1,GADD45A,ICAM1,CEBPB,PANX1,SOCS3,DUSP5,CXCL1,TNFAIP2,KDM6B,IL6, FOSL2,PDE4B,ZFP36,BIRC3,IL15RA,JUNB,NFIL3,TNFAIP3,RCAN1,CXCL2,TSC22D1,NFKB1,OLR1,ETS2,TAP1,SOD2,MCL1,MAP2K3,CCNL1,CEBPD,EGR2,JUN,BTG3, FJX1,CCRL2,GFPT2, LAMB3,BCL3,HBEGF,MAFF,SPHK1,CD83,CDKN1A,ZBTB10,ATF3,IER3,PFKFB3,PTGS2,B4GALT5,DENND5A,PLAU,NINJ1,NFKBIE,RELB,EFNA1,BTG1,BCL2A1, TNIP1,PMEPA1,MSC,CSF1, BCL6,PNRC1,PLK2,NFE2L2,RIPK2,PLAUR,DNAJB4,SLC2A3,PPP1R15A,RELA,IFNGR2,BHLHE40,TRAF1,EIF1,MARCKS,TANK,RNF19B,LITAF,GEM,MXD1,TNIP2,BIRC2,NFKB2,CD44,TRIP10, TGIF1
HEDGEHOG SIGNALING	0.80	LDB1,TLE3,RASA1,L1CAM,AMOT,ETS2,PML,ACHE,MYH9,DPYSL2,NRP2,OPHN1,NRCAM,NRP1,CRMP1

Supplementary table 3. Genes upregulated in liver and thyroid group with high GDF15 expression.

Geneset	-log10(FDR-*q*val)	Gene lists
TNFA SIGNALING VIA NFKB	1.86	TIPARP,SLC2A6,NR4A2,ICAM1,NFKBIE,EGR1,AREG,MXD1,TANK,BTG2,CD44,NR4A1,INHBA,PMEPA1,ATF3,DUSP4,PTX3,BTG1,TNC,YRDC,HBEGF,BMP2,EIF1,CCRL2,MSC,PTGS 2,GPR183, PNRC1,SOCS3,KDM6B,NR4A3,IL1B,CCL20,GADD45A,B4GALT1,TNIP2,CCNL1,BCL2A1,SERPINB2,BCL6,KLF9,IL6,CSF1,TGIF1,TAP1,OLR1,KLF6,PTPRE,ZFP36,TNFAIP6,CLCF1,CXCL10,LIF, NFKB2,ICOSLG,RCAN1,KLF10,RELA,RELB,SQSTM1,SOD2,JUNB,CCL2,TUBB2A,GADD45B,CXCL11,PANX1,B4GALT5,MCL1,MAP2K3,PDE4B,RIPK2,IL15RA,LA MB3,PLAU,SERPINB8, DRAM1,IER3,SERPINE1,G0S2,ZC3H12A,GCH1,BTG3,ACKR3,RNF19B,FJX1,EFNA1,NAMPT,PHLDA1,SLC2A3,SDC4,CXCL2,CEBPD,PFKFB3,BCL3,CXCL3,SPHK1,FOSL2,MYC,LDLR,NFIL3, GEM,CEBPB,GFPT2,MAFF,BHLHE40,SPSB1,CDKN1A,FOSL1,PLAUR
INFLAMMATORY RESPONSE	1.33	SLC7A2,EDN1,CSF3R,OSM,GNAI3,ACVR1B,IL10RA,MET,IRF1,CXCL6,RGS1,CCL7,BDKRB1,ADRM1,ICAM1,CXCL8,CCR7,PIK3R5,CSF3,MXD1,HRH1,TNFSF15,SLC31A2,BTG2,CMKLR1, SLC31A1,INHBA,C3AR1,CXCL9,PROK2,HBEGF,FZD5,ADGRE1,CCRL2,EREG,GPR183,CLEC5A,LCP2,MSR1,IL1B,AHR,CCL20,IL10,LAMP3,TNFRSF1B,IL6,BST2,LPAR1,CSF1,L YN,MARCO, OLR1,KLF6,MEFV,PTPRE,RHOG,GNA15,TNFAIP6,CXCL10,LIF,ICOSLG,IL18RAP,RELA,AQP9,FPR1,CCL2,PVR,IFITM1,CXCL11,CD55,ATP2A2,KCNJ2,PDE4B,RIPK2,SEL ENOS,PTGIR,IL15RA, OSMR,STAB1,PDPN,ADM,IL1R1,SERPINE1,ITGB3,IL18R1,GCH1,TPBG,BEST1,SLC7A1,NAMPT,IL4R,C5AR1,SPHK1,MYC,HIF1A,IRAK2,LDLR,MMP14,ITGA5,TIMP1,CDKN1A,PLAUR
COMPLEMENT	1.26	STX4,LGMN,GNB2,PLAT,PPP4C,PPP2CB,C3,F5,ITGAM,F2,LAMP2,C1QC,CR1,CDA,LAP3,KIF2A,TNFAIP3,GNAI3,ADAM9,CP,IRF1,COL4A2,RABIF,KLK1,DOCK4,ME1,CASP3,ACTN 2,PIK3R5, CD59,GMFB,USP14,FCN1,CA2,APOC1,DOCK10,CTSS,PRSS3,SH2B3,CLU,SRC,C1S,SERPINC1,LGALS3,SERPINA1,PREP,PIK3CA,LRP1,GZMB,LCP2,LTF,C1R,FN1,DUSP6, SERPINB2,PFN1,IL6, SERPING1,LYN,CTSC,OLR1,DGKH,S100A12,GNB4,RHOG,CTSL,C2,PLG,FCER1G,CFH,CFB,ANXA5,S100A9,MMP8,CD55,DPP4,PLA2G4A,SERPINE1,CTSB,CASP4,CASP5,HSPA5,CBLB, PLSCR1,CEBPB,MMP14,MAFF,PIM1,TIMP1,PLAUR
HYPOXIA	1.20	ENO3,PCK1,DTNA,IGFBP1,CXCR4,CA12,ALDOA,GPI,SULT2B1,TNFAIP3,PGM1,PDK3,CPKLHL24,PGM2,SLC2A1,COL5A1,PFKP,PKP1,TIPARP,TPI1,ETS1,PAM,ISG20,TKTL1,ATF3,BTG1, GPC1,LALBA,NOCT,GAPDH,PNRC1,BGN,DDIT4,TES,PHKG1,ERRFI1,MYH9,IL6,SLC2A5,KLF6,SDC3,ANGPTL4,ZFP36,PGK1,ANXA2,LXN,NDRG1,MT2A,ERO1A,AKAP12,GP C4,PDK1, TGFB3,UGP2,SIAH2,PLIN2,GBE1,TGFBI,EDN2,P4HA1,STBD1,HMOX1,STC1,ADM,IER3,SERPINE1,STC2,HAS1,ACKR3,ENO1,HSPA5,JMJD6,TPBG,KDELR3,GLRX,EFNA1,LDHA,MEM45A, P4HA2,DDIT3,AK4,SLC2A3,SDC4,PFKFB3,FOSL2,NFIL3,MAFF,PIM1,BHLHE40,CDKN1A,PLAUR
APOPTOSIS	1.15	BNIP3L,CD14,HSPB1,RHOB,PRF1,PLPPR4,PLAT,SAT1,WEE1,ROCK1,F2,GSR,SMAD7,IRF1,PPP3R1,GUCY2D,BCL2L1,FEZ1,EGR3,DPYD,CASP3,LMNA,CTNNB1,BMF,BTG2,ISG20,CD44,LEF1, TOP2A,ATF3,F2R,CLU,LGALS3,SC5D,IFNGR1,BMP2,IGF2R,NEDD9,EREG,BGN,ETF1,IL1B,DNAJA1,GADD45A,IL6,KRT18,TAP1,EMP1,CCNA1,DNAJC3,HGF,GNA15,M MP2,CCND2,BID, RELA,SQSTM1,SOD2,IFITM3,TGFB2,GADD45B,PEA15,LUM,MCL1,CDK2,HMOX1,ANKH,IER3,PMAIP1,CASP4,GCH1,BTG3,RARA,TNFRSF12A,BAX,DDIT3,SLC20A1,ANXA1,TIMP1,FAS, CDKN1A
EPITHELIAL MESENCHYMAL TRANSITION	1.10	ACTA2,NOTCH2,CAP2,PMP22,CADM1,VCAM1,RHOB,CALD1,SGCB,MYL9,TGM2,SLC6A8,SAT1,FBLN2,RGS4,BDNF,SFRP1,SPP1,MSX1,SGCG,TNFAIP3,FBN2,COPA,PLOD2,COL4A2, FERMT2,CXCL6,FOXC2,COL5A1,DPYSL3,LAMA3,MXRA5,P3H1,BASP1,PLOD1,CXCL8,PRRX1,WIPF1,COL6A2,SNTB1,AREG,CD59,PCOLCE2,PRSS2,LOXL2,GJA1,FBLN1,CD44,FSTL1,ITGB5, SERPINH1,INHBA,LAMC2,IGFBP2,PMEPA1,PTX3,TAGLN,GPC1,TNC,COL6A3,LRRC15,MMP1,SFRP4,BGN,LRP1,DAB2,FN1,FLNA,GADD45A,FSTL3,COL4A1,IL6,C DH11,ITGB1,BMP1, PLOD3,ITGA2,PPIB,ECM1,TGFB1,MMP2,CTHRC1,NTM,IGFBP4,PVR,FGF2,FAP,GADD45B,TGFBI,LUM,COL8A2,COLGALT1,GREM1,SERPINE1,ITGB3,GLIPR1,THBS1,TNFRSF12A,ITGAV, CALU,COL12A1,SDC4,PDLIM4,VCAN,IL32,NNMT,GEM,TPM4,MMP14,SERPINE2,MGP,ITGA5,TIMP1,FAS,THBS2,PLAUR
IL6 JAK STAT3 SIGNALING	1.06	MAP3K8,CD14,TLR2,CBL,BAK1,CRLF2,IRF9,CSF3R,ACVR1B,IRF1,CCL7,IL1R2,PIK3R5,PTPN1,IL3RA,CD44,MYD88,CXCL9,STAT2,IFNGR1,REG1A,STAT1,CCR1,PLA2G2A,SOCS3,IL1B, IL13RA1,TNFRSF1B,IL6,IL2RA,CSF1,LTBR,CXCL10,TGFB1,TNFRSF21,CXCL11,IL15RA,HMOX1,LEPR,OSMR,IL1R1,ITGB3,IL18R1,TNFRSF12A,IL4R,TNFRSF1A,STAT3,CXCL3,PIM1,FAS
MTORC1 SIGNALING	1.03	SLA,PSMC6,PSMC4,IMMT,TFRC,TMEM97,USO1,CANX,LGMN,ACSL3,PHGDH,RDH11,SCD,HMGCR,CXCR4,GCLC,ALDOA,GPI,PPIA,GSR,PSMD12,SYTL2,NFKBIB,PSMB5,TXNRD1, PSMA4,SERP1,PGM1,IFI30,GLA,PLOD2,CDC25A,EEF1E1,G6PD,M6PR,SLC2A1,TBK1,HPRT1,TOMM40,ME1,TPI1,PSMD14,TUBG1,PSMA3,RIT1,RRM2,BTG2,ACLY,IFRD1,CCT6 A,SERPINH1, PNO1,HSPD1,ACACA,HSPE1,PPA1,CYP51A1,MLLT11,RPN1,SC5D,EDEM1,HSPA4,GAPDH,FKBP2,DDIT4,ARPC5L,TES,CALR,ETF1,RAB1A,DHCR7,GOT1,HMGCS1,I DI1,UBE2D3,DDX39A, ABCF2,CTSC,STIP1,SLC7A11,PGK1,BUB1,SQLE,ERO1A,PDK1,SQSTM1,SDF2L1,FADS2,GBE1,NUPR1,ATP2A2,MAP2K3,P4HA1,HSPA9,HSP90B1,STC1,SLC 1A4,MTHFD2,ENO1,PSAT1,HSPA5, TUBA4A,INSIG1,FADS1,ASNS,EGLN3,BCAT1,EIF2S2,GLRX,LDHA,NAMPT,DHCR24,DDIT3,AK4,PITPNB,SLC2A3,ACTR3,SLC7A5,XBP1,SLC1A5,ELOVL5,STARD4,LDLR,NFIL3,SHMT2,TRIB3, BHLHE40,PNP,CDKN1A
COAGULATION	0.97	PROZ,LGMN,GNB2,PLAT,C3,HMGCS2,F2,LAMP2,MMP10,ADAM9,DUSP14,RABIF,MMP11,FURIN,F9,FGA,APOC1,C8A,CLU,WDR1,HRG,C1S,SERPINC1,CFI,MBL2,PROS1,SERPI NA1,FGG, MMP1,PREP,F11,APOC2,LRP1,F2RL2,C1R,FN1,DUSP6,CPN1,SERPINB2,CTSE,CFD,SERPING1,OLR1,BMP1,ARF4,ITGA2,MMP2,C2,PLG,CFH,CFB,MMP8,DPP4,PLAU,SE RPINE1,ITGB3,CTSB, THBS1,MMP14,MAFF,ANXA1,TIMP1,F12
P53 PATHWAY	0.90	PPM1D,NHLH2,PERP,PHLDA3,TNNI1,ST14,ACVR1B,IFI30,MKNK2,CDKN2A,VDR,ADA,DDB2,RAP2B,MXD1,FBXW7,BTG2,TAX1BP3,ATF3,F2R,BTG1,PVT1,KRT17,POLH,OSGIN1,HBEGF, BMP2,TCHH,RHBDF2,PITPNC1,DDIT4,GADD45A,CLCA2,SERPINB5,ALOX15B,SERTAD3,TAP1,IRAK1,EPHA2,PTPRE,CDKN2B,HSPA4L,SLC7A11,AEN,NDRG1,TGFB1,LIF,CCND2,STOM, PLK3,ABHD4,NUPR1,SLC3A2,STEAP3,MDM2,VWA5A,HMOX1,SLC19A2,SEC61A1,DRAM1,IER3,RRAD,PROCR,RPS27L,RNF19B,BAX,DDIT3,SPHK1,ZMAT3,SFN,ZFP36L1,TRIB3,UPP1,FAS, CDKN1A,INHBB,PDGFA

Supplementary table 4. Clinical and biochemical characteristics of study subjects according to serum GDF-15 levels.

	Serum GDF15 (n = 162)		*P* value
	Low (n = 81)	High (n = 81)	
GDF15 (pg/mL)	281.5 (193.8-379.1)	730.0 (555.5-1012.7)	<0.001*
Age (years)	41.7 ± 11.7	43.9 ± 12.2	0.236†
Gender (female)	62 (76.5)	56 (69.1)	0.289‡
BMI (kg/m^2)	23.1 ± 3.4	24.8 ± 3.7	0.005†
SBP (mmHg)	117.0 (111.5-128.5)	129.0 (118.0-137.0)	0.001*
DBP (mmHg)	78.0 (73.0-86.0)	82.0 (75.0-87.5)	0.140*
Fasting glucose (mg/dL)	95.0 (90.0-101.0)	100.0 (92.5-107.5)	0.005*
AST (IU/L)	17.0 (14.0-21.0)	18.0 (16.0-23.0)	0.063*
ALT (IU/L)	14.0 (10.0-18.5)	20.0 (12.0-28.5)	<0.001*
Alkaline phosphatase (IU/L)	58.0 (47.0-69.0)	64.0 (51.5-83.5)	0.010*
Total bilirubin (mg/dL)	0.5 (0.4-0.7)	0.6 (0.5-0.9)	0.012*
TSH (mIU/mL)	1.37 (0.92-1.80)	1.45 (0.95-2.30)	0.193*

GDF-15, growth differentiation factor-15; BMI, body mass index; SBP, systolic blood pressure; DBP, diastolic blood pressure; AST, aspartate transaminase; ALT, alanine transaminase; TSH, thyroid stimulating hormone.

*P values calculated by Mann-Whitney-U test. Data are expressed as the median and inter-quartile ranges (IQR).

† P values calculated by Student's t-test. Data are expressed as the mean ± standard deviation (SD).

‡ P values calculated by $\chi 2$ test. Data are expressed as numbers and percentages (%).

Supplementary table 5. Pearson's correlation coefficient and Spearman's rho between the differences in serum GDF-15 levels (log2) and the differences in various parameters

	Pearson's coefficient (*r*)	*P* value	Spearman's rho (*r*)	*P* value
BMI (kg/m^2)	0.332	<0.001	0.292	<0.001
SBP (mmHg)	0.214	0.006	0.255	0.001
DBP (mmHg)	0.072	0.364	0.152	0.054
Fasting glucose (mg/dL)	0.289	<0.001	0.301	<0.001
ALT (IU/L)	0.228	0.003	0.292	<0.001
ALP (IU/L)	0.311	<0.001	0.300	<0.001
Total bilirubin (mg/dL)	0.283	<0.001	0.241	0.002

GDF-15, growth differentiation factor-15; BMI, body mass index; SBP, systolic blood pressure; DBP, diastolic blood pressure; ALT, alanine transaminase; ALP, Alkaline phosphatase.
